# The abscopal effect in patients with cancer receiving immunotherapy

**DOI:** 10.1016/j.medj.2023.02.003

**Published:** 2023-03-08

**Authors:** Blessie Elizabeth Nelson, Jacob J. Adashek, Steven H. Lin, Vivek Subbiah

**Affiliations:** 1 Department of Investigational Cancer Therapeutics, The University of Texas MD Anderson Cancer Center, Houston, TX 77030, USA; 2 Department of Oncology, The Sidney Kimmel Comprehensive Cancer Center, The Johns Hopkins Hospital, Baltimore, MD, USA; 3 Department of Radiation Oncology, The University of Texas MD Anderson Cancer Center, Houston, TX 77030, USA

## Abstract

Interest in the abscopal effect has been rekindled over the past decade with the advent of immunotherapy. Although purportedly elusive, this phenomenon is being increasingly reported. Venturing further using a multimodality approach with an array of systemic agents and unconventional modalities is direly needed. In this perspective, we describe the fundamentals of abscopal responses (ARs), explore combinations with systemic therapies that hold promise in eliciting ARs, and reconnoiter unconventional modalities that may induce ARs. Finally, we scrutinize prospective agents and modalities that exhibit preclinical ability to elicit ARs and discuss prognostic biomarkers, their limitations, and pathways of abscopal resistance for reproducibility.

## SETTING THE STAGE FOR THE ABSCOPAL PHENOMENON

Abscopal is defined as “at a distance from the irradiated volume but within the same organism.” It is derived from “ab-,” with the denotation “position away from,” and the Latin word “scopus,” which means “mark or target for shooting at.” The term abscopal was coined by R.H. Mole in 1953.^1^

Historically, the effectiveness of radiotherapy (RT) for tumors was believed to result from direct cytotoxic effects and DNA damage. However, numerous case reports and case series have demonstrated an effect distant from the irradiated area.^1,2^ Initial work on the abscopal effect stemmed from the inherent lymphopenia caused by radiation and the intimate interaction between RT and the immune system.^3^ The abscopal effect recently returned to the forefront of eliciting systemic responses with the emergence of immunotherapy as one of the pillars of oncology. Although rare, authors have reported abscopal effects in patients with multiple tumor types, such as melanoma, lymphoma, and renal cell cancer. Also, preclinical studies have shown that irradiated tumor cells express neoantigens via major histocompatibility complex I and thus present to CD8^+^ T cells, activating the immune cascade. More specifically, this interaction is facilitated by Toll-like receptor 4 activation.^4^ The majority of reported clinical work on abscopal effects is from anecdotal case reports. However, as the abscopal effect continues to gain mainstream acceptance, more prospective studies are underway to use this potential benefit. The TARGIT-A study, in which the efficacy of intraoperative RT was compared with external beam RT in patients with breast cancer, showed that those who underwent lumpectomy and received either intraoperative or external beam RT had no added benefit with external beam RT (hazard ratio [HR], 0.96 [95% confidence interval (CI), 0.68–1.35]; p = 0.8).^5^ However, in patients who received intraoperative RT, the rate of local recurrence had no effect on distant metastasis, and they had fewer non-breast-cancer-related deaths, which was thought to result in part from an abscopal effect (HR, 0.38 [95% CI, 0.17–0.88]; p = 0.0093).^5^

**Figure 1.**
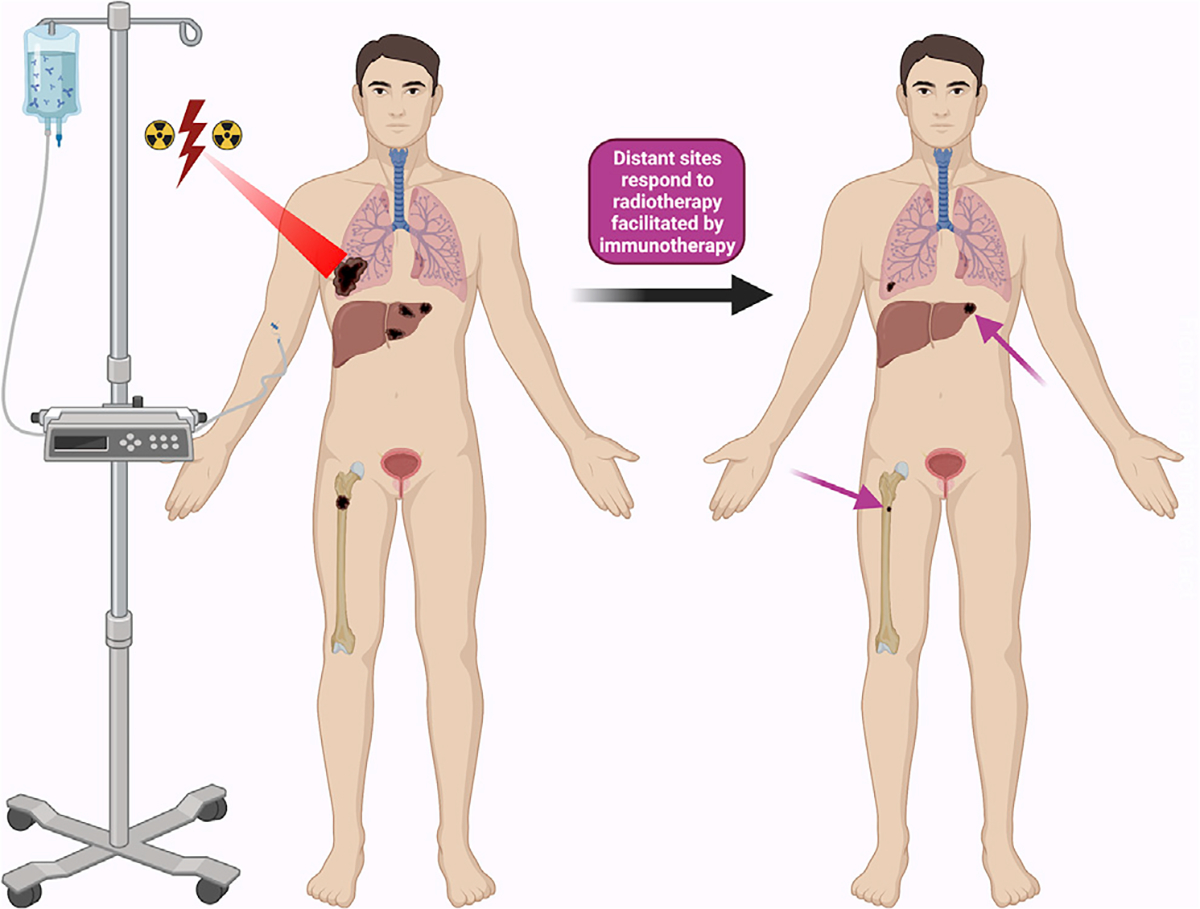
Clinical effect of the abscopal phenomenon The abscopal phenomenon is a systemic response seen with a primary tumor is irradiated or combined with immunotherapy that may stimulate an immune response against tumors outside the radiation field at distant sites.

The seminal preclinical work on how radiation elicits abscopal effect stemmed from a study led by Demaria et al. in which investigators irradiated one of two murine mammary tumor models combined with treatment with growth factor Flt3-ligand (Flt3-L), which exclusively elicited abscopal responses (ARs) in the unirradiated tumor in the presence of RT.^6^ Since then, a similar preclinical investigation conducted by Dewan et al. in murine mammary models utilized a combination of anti-cytotoxic T-lymphocyte-associated antigen 4 antibody and radiation, showing AR with statistical significance in the unirradiated lesions.^7^ These studies have served as the basis for continued efforts to identify the abscopal effect in patients with cancer as well as to validate the clinical efficacy of multimodality therapy and effect. Because the abscopal effect continues to be deliberated among the oncology community, in this review, we explore this rapidly emerging phenomenon (Figure 1).

## DECIPHERING THE ABSCOPAL RESPONSE IN CLINICAL TRIALS

Some phase 2 and 3 trials have demonstrated ARs. Overall survival (OS) durations attributed to the abscopal effect are listed in Table 1.

One of the earliest seminal trials demonstrating the abscopal effect involved patients with multiple tumor types receiving granulocyte-macrophage colony-stimulating factor (GM-CSF; 125 mg/m), which is known to be an immunoadjuvant, especially in the setting of RT, that facilitates AR.^8^ Investigators administered GM-CSF subcutaneously along with radiation and their choice of chemotherapy, noting an AR rate of 27%. They also saw improved OS in patients who had ARs, with a median OS duration of 21 months, compared with a median duration of 8.3 months in non-responders.

Chicas-Sett et al.^9^ performed a systematic review of the literature, looking at stereotactic radiosurgery (SRS)/stereotactic body RT (SBRT) and ipilimumab and the incidence of ARs in advanced tumors. In 16 retrospective or randomized studies, the median abscopal effect incidence rate was 26.5%, with improved patient outcomes attributable to ARs shown to be a clinically relevant phenomenon. Specifically, the researchers saw improved clinical outcomes in patients given RT at greater than 3 Gy/fraction along with ipilimumab.

A pooled analysis of two randomized trials—the phase 2 PEMBRO-RT trial and phase 1/2 MDACC trial—examined the role of radiation in ARs to immunotherapy in patients with advanced lung cancer.^10^ Both trials enrolled patients with metastatic non-small cell lung cancer and at least one unirradiated tumor to assess ARs. In the PEMBRO-RT trial, the sequence of administration of the first dose of pembrolizumab was at the end of irradiation (24 Gy in 3 fractions), and in the MDACC trial, pembrolizumab was given simultaneously with radiation (50 Gy in 4 fractions or 45 Gy in 15 fractions). The AR rate was 19.7% in the single-agent pembrolizumab arm but 41.7% in the doublet pembrolizumab and RT arm (p = 0.0039) in the pooled analysis of the above 2 trials. The abscopal disease control rate was 43.4% for pembrolizumab and 65.3% for pembrolizumab and RT (p = 0.0071). Furthermore, the pembrolizumab monotherapy arm had a median progression-free survival duration of 4.4 months, whereas the pembrolizumab plus RT arm had a median duration of 9 months (p = 0.045). The median OS durations were 8.7 months for pembrolizumab only and 19.2 months for pembrolizumab and RT (p = 0.0004).

Use of traditional irradiation techniques is not required to produce abscopal effects. Tubin et al.^11^ developed the SBRT-based partial tumor irradiation method targeting the hypoxic parts of bulky tumors (SBRT-PATHY). This method induces an abscopal effect because it induces a radiation-induced bystander effect by manipulating tumor hypoxia, protecting the peritumoral environment, and partial irradiation of the bulky tumor. Tubin and colleagues performed a phase 2 trial in which they used the SBRT-PATHY technique in 60 patients with bulky non-resectable non-small cell lung cancer (SBRT-PATHY, chemotherapy, and palliative RT arms). The 1 year OS rate was highest in the SBRT-PATHY cohort at an impressive 75% (p = 0.099). Also, the primary irradiated tumor control rate was 95% in the SBRT-PATHY cohort compared with only 20% in the other two cohorts. The investigators observed bystander effects in 95% of patients and ARs in 45% of the SBRT-PATHY cohort.^11^

To build on this clinical evidence, multiple ongoing clinical trials are accruing subjects (Table 2). These trials include those examining combinations of growth factors, immune checkpoint inhibitors, and chemotherapy. Many of these trials are tumor agnostic, with researchers testing the effect of concurrent or sequential neoadjuvant therapy with RT and a portfolio of immunotherapeutic agents.

## TANGIBLE EVIDENCE OF THE ABSCOPAL PHENOMENON

One of the challenges restricting the viability of effective cancer therapy is cancer heterogeneity across the diverse spectrum of humans and their tumor phenotypes. Conventional methodologies of targeting adaptive immunity by using personalized neoantigen vaccines have not been very effective. To address this deficit, Min et al.^12^ created antigen-capturing nanoparticles (AC-NPs) to effectively engulf the tumor-derived protein antigens released during RT and present them to antigen-presenting cells to activate the immune system. The authors formulated the NPs using a poly(lactic-co-glycolic) acid base and incubated them with irradiated B16F10 melanoma cells. To detect the neoantigen tumor burden, *in silico* analysis revealed quantification of the antigen production by mass spectrometry and captured damage-associated molecular patterns and HMGB1, which are known to potentiate ARs. In mice with flank B16F10 melanomas,^12^ Min and colleagues administered anti-programmed cell death protein 1 (PD-1) therapy with irradiation of primary tumors and monitored ARs in secondary tumors. The combination of RT, anti-PD-1 therapy, and treatment with poly(lactic-co-glycolic) acid AC-NPs resulted in a complete AR rate of 20% that was durable even at 3 months of follow up. Administration of AC-NPs and radiation mechanistically increased cellular uptake of AC-NPs for delivery of neoantigens, eliciting a robust adaptive immune response. This approach holds great promise for precision irradiation and abscopal delivery of systemic response and can be rapidly translated to the clinic.^12^

In the same vein, researchers studied NBTXR3 prior to surgery in patients with soft tissue sarcoma in a phase 2 trial.^13^ NBTXR3 is a first-in-class agent that is a radiosensitizer with hafnium oxide NPs. The investigators observed a 16% complete pathological response rate in 14 patients in an NBTXR3 and RT cohort compared with 8% in an RT-alone cohort (p = 0.044).^13^ Hu et al.^14^ took NBTXR3 one step further and explored its synergy with localized irradiation in a mouse model of lung cancer with anti-PD-1 therapy resistance. They noted that both programmed death-ligand 1-sensitive and -resistant mice given a combination NBTXR3 with anti-programmed death-ligand 1 therapy exhibited disease regression, with significant ARs in secondary tumors along with enrichment of the T cell receptor repertoire. Currently, a phase 1/2 clinical trial (ClinicalTrials.gov: NCT05039632) is exploring NBTXR3 along with irradiation and treatment with ipilimumab and nivolumab in patients with advanced solid tumors with lung and/or liver metastases.

Additionally, Woo et al.^15^ showed that the stimulator of interferon genes mechanism can be upregulated by neoantigen presentation, triggering dendritic cell activation, presentation to CD8 T cells, type I interferon production, and tumor microenvironment infiltration of T cells. Based on this, Conde et al.^16^ combined chimeric antigen receptor T cell therapy and treatment with the immunomodulatory agent 2′3′-cGAMP in preclinical murine melanoma models to improve tumor microenvironment modulation and enhance epitope spreading with stimulator of interferon genes-L/ligand. This led to tumor growth suppression and improved OS in these murine models, with abscopal effects on distal non-injected lesions. Importantly, stimulator of interferon genes signaling and dendritic cells exclusively activated epitope spread and the anti-tumor effects of the combination therapy. This finding elucidates the potential of 2′3′-cGAMP as a potent radioimmunoadjuvant, as it does not lead to T cell death and synergizes with chimeric antigen receptor T cell therapy to elicit ARs without the need for RT, especially in solid tumors for which cellular therapy has yet to gain ground.

Another key goal in chasing the abscopal phenomenon is the ability to quantitatively predict and measure its effects. Researchers are developing radiodiagnostic biomarkers to capture and quantify tumor response to systemic therapy and radiation and hence aid in patient selection for effective therapies and, in turn, understand prognosis for survival outcomes. Sun et al.^17^ explored this question to demonstrate associations of patient outcomes in advanced cancer with the combination of immunotherapy and RT by investigating radiomic data obtained from computed tomography scans with contrast and infiltration of CD8 T cells. Of 99 patients with multiple tumor types in this retrospective analysis, 97% underwent hypofractionated RT with a median dosing schedule of 8 Gy in three fractions and a median of four cycles of immunotherapy. 23% of irradiated lesions responded to the given therapy, while 17% of non-irradiated lesions demonstrated response. In 91 patients evaluable for AR, the AR rate in distant lesions was 26%, and patients with ARs had a significantly higher OS than did patients without ARs (HR, 0.31; p = 0.027). Increased median CD8 radiomic score for responding lesions was also noted (p = 0.0066). Hence, Sun and colleagues concluded that lesions with increased baseline CD8 T cell radiomic scores had improved responses upon follow-up computed tomography with or without irradiation, accounting for tumor heterogeneity in this study, which included colorectal, melanoma, lung, bladder, and head and neck cancers. Hence, identification of “cold” or “hot” tumors by analyzing the biological radiomic signature of CD8 T cells may ensure delivery of radiation to the appropriate immunogenic lesion and thus increase tumor antigen production. Translating the results of this study to prospective randomized trials to predict AR and choose the right lesion for local therapy that will elicit response holds great promise for precision medicine to harness abscopal effects.

Yang and colleagues hypothesized that intercellular adhesion molecule 1 (ICAM-1; CD54), which is known for stabilizing cell-cell interactions and facilitating endothelial leukocyte transmigration, is highly expressed in non-irradiated tumors reactive to RT.^18^ They used quantitative proteomic analysis to assess non-irradiated tumors for ICAM-1 expression. These lesions demonstrated abscopal effects elicited by in-field RT for primary lesions. The response of non-irradiated tumors correlated with increased expression of ICAM-1. Mechanistically, RT increased ICAM-1 expression in CD8^+^ T cells, proving that the abscopal effect of RT is T cell pathway dependent. The same authors also noted that treatment with imiquimod, which is an immune modulator activating Toll-like receptor 7 (TLR-7), synergized with RT to suppress the growth of non-irradiated lesions via the abscopal effect. Hence, use of ICAM-1-targeted positron emission tomography provides improved non-invasive monitoring and assessment of ARs resulting from combination treatment modalities. In addition, targeting ICAM-1 by combining treatment with TLR-7 agonists and RT holds clinical promise in generating ARs.^19^

Poly (ADP-ribose) polymerase (PARP) proteins participate in DNA damage response, and researchers have explored PARP inhibitors for radiosensitization of tumors and in combination with RT and immunotherapy. PARP inhibitors can induce synthetic lethality in tumors that are homologous recombination deficient and block DNA repair and replication, leading to tumor suppression. Hence, targeting the PARP pathway increases the occurrence of DNA double-strand breaks and synergizes with radiation. RT and PARP inhibition together will potentiate immunogenic cell death, which can intensify response to RT with concurrent immunotherapy.^20^ Translating this in clinical practice in patients with metastatic ovarian cancer, the phase 1/2 MEDIOLA trial (ClinicalTrials.gov: NCT02734004) looked at treatment with durvalumab and olaparib, which led to a 71.9% overall response rate in patients with germline BRCA-mutant, platinum-sensitive tumors. In comparison, the phase 1/2 TOPACIO trial (ClinicalTrials.gov: NCT02657889) combined pembrolizumab with niraparib for treatment of advanced or metastatic triple-negative breast cancer or recurrent ovarian cancer, producing an overall response rate of 25%. Regarding breast cancer, investigators in three phase 1/2 clinical trials (ClinicalTrials.gov: NCT04683679, NCT04690855, and NCT04837209) are exploring combinations of immunotherapy and hypofractionated RT. This concept is also being explored in the PRIO trial (ClinicalTrials.gov: NCT04728230), in which olaparib and durvalumab are combined with carboplatin, etoposide, and/or RT in treatment of extensive-stage small-cell lung cancer. Exploring the results of these trials will show whether PARP inhibitors hold potential for eliciting ARs.

Although ARs are frequently explored in patients with solid tumors, researchers in a prospective phase 2 trial are currently examining the abscopal effects of radiation and nivolumab in 29 patients with relapsed/refractory Hodgkin’s lymphoma (ClinicalTrials.gov: NCT03480334) and performing correlative studies to evaluate various parameters for anti-PD-1 therapy and RT. Specifically, the investigators are combining 240 mg nivolumab once every 2 weeks and RT at 20 Gy in 10 fractions starting on day 6 of nivolumab administration. Results of this trial are expected by December 2024.^21^

## MECHANISMS OF RADIORESISTANCE IN PATIENTS WITH ABSCOPAL EFFECT

Tumor hypoxia is a well-established intermediary of radioresistance. Targeting the oxidation phosphorylation demand of cells can decrease tumor oxygen demand and in turn effectively modulate the tumor microenvironment.^22^ Atovaquone is an anti-protozoal drug that inhibits electron transport in mitochondria, resulting in inhibition of synthesis of nucleic acids and ATP, which in turn assuages tumor hypoxia and increases tumor radiosensitization.^23^ Combining atovaquone with treatment of classically hypoxia-driven tumors may be a fruitful strategy in improving hypoxic conditions in tumor for effective tumoricidal killing.

The abscopal effect can be hampered by immunosuppressive effects of the tumor microenvironment. Contributing factors are low immunogenicity of released neoantigens, immunosuppressive cytokines, and the presence of immunosuppressive cells in the tumor microenvironment, such as regulatory T cells and myeloid-derived suppressor cells. One such example of T cell exhaustion was seen in a study combining RT with treatment with the anti-cytotoxic T-lymphocyte-associated antigen 4 antibody ipilimumab for metastatic melanoma.^24^ This study demonstrated that the combination resulted in increased expression of programmed death-ligand 1 in tumor cells and thus T cell fatigue, leading to a stunted abscopal effect. Researchers observed another mechanism of resistance in hypoxia-driven tumors, which increased the recruitment of regulatory T cells and led to an immunosuppressive tumor microenvironment.^25,26^ Authors have widely documented hyperprogression of disease in patients in multiple cancer histologies who have undergone immune checkpoint blockade.^27,28^ However, the literature has a paucity of case reports demonstrating hyperprogression of disease following RT and immunotherapy.^29^

## WHY IS THE ABSCOPAL EFFECT NOT EASILY REPRODUCIBLE?

As with every phenomenon studied, the rate-limiting steps in achieving an optimal AR must be considered. Although extensive preclinical data support the abscopal phenomenon, the translational strength of preclinical mouse studies of abscopal effects with multimodality therapies is still underwhelming and requires corroboration. One reason for the inability to achieve reproducibility of ARs is likely heterogeneity of tumor and immunological phenotypes in clinical practice.

Damage to the structural tumor environment that occurs with varying radiation doses must be examined. For example, Park et al.^30^ looked at the vascular disruption in the tumor microenvironment that occurs when SBRT or SRS is administered. This alteration in the angiogenic environment can alter the cascade of events required to elicit AR and facilitate synthetic lethality of drug bioavailability in tumor tissue.

Paramount to understanding and validating AR is the advancement of reliable biomarkers that can be used to consistently track the level of neoantigen burden and presentation and immune effector stimulation. Developing a prognostic system for predetermination of which patient populations, histologies, and/or combination strategies will enable precise harnessing of ARs and hence avoid unnecessary after-effects of a “shot-gun” approach and enable prediction of efficacy responses via the abscopal effect.^31^

Another important factor to consider in patients with intracranial metastasis is how molecular mediators of abscopal effects can effectively cross the blood-brain barrier, which varies according to the organs targeted. Ishiyama et al.^32^ reported a case of metastatic renal cell carcinoma with progression of brain metastases while experiencing complete ARs in pulmonary and lymph node lesions after SBRT to the bone and spine (40 Gy in five fractions) and SRS of the brain (18 Gy in one fraction). However, when the integrity of the blood-brain barrier is disrupted, mediators of AR can be transported across it. Gui et al.^33^ proposed that oxidative stress, imbalance among metabolites and signaling molecules, and inflammation play roles in disrupting the blood-brain barrier. This disruption results in permeability of the barrier to tumor-associated antigens, which play a crucial role in immune effector stimulation and hence increasing the probability of AR.

Sequencing of immunotherapy and RT is paramount to eliciting abscopal effects, and exploring the window of opportunity for optimal synthetic lethality of RT and with immunotherapy requires further corroboration. Sixty-two patients with metastatic squamous cell cancer of the head and neck underwent treatment with nivolumab in a phase 2 randomized trial.^34^ They were randomized to receive concurrent SBRT (27 Gy in three fractions) and nivolumab or nivolumab alone. The difference in lesion ARs in the SBRT and nivolumab (34%) and nivolumab-alone (29%) groups was not significant (p = 0.86). Of note is that nivolumab was administered concurrently with radiation unlike in the PEMBRO-RT trial, in which immunotherapy was administered sequentially after SBRT. Both trials did demonstrate tolerable side effects of combination modalities. Further studies are required to elucidate the safety and optimal timing in combining immunotherapy and RT.

The molecular mechanism believed to contribute to the most significant abscopal effects is neoantigen expression.^35^ Death of an irradiated tumor releases substantial necrotic tissue and particles that are collected by antigen-presenting cells and thus presented to the CD8^+^ T cells.^36^ Furthermore, irradiated tissue is believed to release of damage-associated molecular patterns, which further enhance the immune response.^37^ Work on how to combine agents that stimulate or increase the activation of antigen-presenting cells after delivery of radiation is continuing.^38,39^

The level of immunogenic cell death is directly proportional to the burden of neoantigen shedding from tumor sites for presentation of tumor antigens by antigen-presenting cells.^40^ Controlling and harnessing the priming process of immune cells against tumor antigens is important to reproducing abscopal effects effectively.

Although researchers have seen exciting responses regarding abscopal effects with combinations of different therapies with RT, identification of responders to such therapy is limited, and future questions to address these deficits are depicted in Table 3.^41^ Biomarker validation and prognostication markers for elicitation of AR are areas of active research.

## A WORD OF CAUTION

In a crucial pediatric study, Diallo et al.^42^ examined the frequency of secondary tumors in previously irradiated fields and the correlation with the radiation dose delivery. Of 115 secondary tumors, 25 were in distant regions, defined as more than 5 cm away from the irradiated tumor volume. The most frequent secondary tumor types were sarcomas (25%), brain and other nervous system tumors (25%), and thyroid carcinomas (16%). The authors noted that the peak secondary tumor distribution was 31% in fields that received less than 2.5 Gy, which raises the question of whether secondary cancers can occur abscopally.^42,43^

## FUTURE DIRECTIONS AND CONCLUDING REMARKS

Understanding the underlying molecular mechanisms that elicit the abscopal effect is key to harnessing this effect. Development of biomarkers for predicting ARs remains elusive. Preclinical and mechanistic rationales for how molecular abscopal mechanisms occur are being investigated and are pending clinical validation. Designing and performing clinical trials with feasible biomarkers and prognostic trackers for the abscopal effect and appropriate patient selection will eventually result in greater clinical relevance of this effect.

Strigari et al.^44^ considered the p53 gene to be a crucial intermediary for abscopal effects. Others conceptualized that the presence of antibodies against calreticulin, heat shock protein 70, tumor necrosis factor alpha, interleukin-18, or NY-ESO-1 or other cytokines after RT can identify patients who are susceptible to ARs.^45^

Surveilling cytokine production, radiomics, and immune priming to measure the burden of neoantigen production may be used to optimize the choice of RT modality, tumor histology, immunogenic status of tumors, and irradiation schedule and to selectively minimize bystander/normal tissue abscopal effects. It would also provide clarity in sequencing of appropriate systemic agents to achieve the best possible abscopal outcomes in modulating the tumor environment. Combining local RT modalities with immune-priming agents and administering them with systemic immune-modulating agents would enhance the abscopal effect. Importantly, the intratumoral phenotype and heterogeneity interplay with response to secondary to tumor metabolism, hypoxia, genomic instability, proliferation, and intrinsic radiosensitivity must be accounted for, as these factors can become barriers to consistent elicitation of ARs.

Among the ever-changing methods of oncological management, multimodality RT approaches, including local and systemic treatments, are more consistent and have tolerable side effects. The effects of novel systemic agents on systemic responses to irradiation remain to be explored. Hence, studies of these effects will add to the abscopal armamentarium.

The serendipitous discovery of the abscopal phenomenon in patients with cancer has revolutionized and harnessed the power of irradiation and other treatment modalities employed with systemic agents more than ever in the treatment of various immunogenic tumors. Although eliciting a consistent, reproducible response remains a challenge, we are ever closer to understanding the complex underlying mechanisms and pathophysiology in the tumor microenvironment required to induce abscopal effects. Exploiting systemic agents with RT modalities is the next step in harnessing the power of the abscopal phenomenon and eventually achieving validation of abscopal effects in daily clinical practice.

## Figures and Tables

**Table 1.** The characteristics of phase 2 and 3 clinical trials and their corresponding OS durations and abscopal response frequencies in patients with the abscopal effect

Clinical trial	Phase	Tumor type	RT modality	Sequence of systemic therapy	RT	Systemic therapy	OS duration	AR frequency (%)

NCT02474186 ^8^	2	multiple	conventional	sequential GM-CSF and concurrent chemotherapy	35 Gy/10 fractions	chemotherapy, GM-CSF	20.98 months	27
ChiCTR-1900027768^46^	1	lung cancer	SBRT	sequential	40–50 Gy/5 fractions and hypofractionated brachytherapy	nivolumab	NR	45.2
NCT03332069 ^47^	3	cervical cancer	conventional	concurrent	50 Gy/25 fractions, brachytherapy, and electrohypothermia	cisplatin	not assessed	24.1
NCT02239900 ^48^	2	multiple	SBRT	concurrent vs. sequential	50–60 Gy/4–10 fractions	ipilimumab	NR	26
NCT03450967 ^49^	2	head and neck squamous cell carcinoma	proton therapy	concurrent	25 Gy/5 fractions	durvalumab and tremelimumab	6.40 months	27.3
NCT02710253 ^50^	2	multiple	conventional or SBRT	concurrent	unknown	immunotherapy	not assessed	11
NCT01502293 ^51^	2	melanoma	electroporation	sequential	six sessions, 1,500 V/cm on days 1, 5, and 8 every 90 days	intratumoral tavokinogene telseplasmid	NR	29.2
NCT02492568; NCT02444741^10^	2; 1/2	lung cancer	conventional or SBRT	concurrent vs. sequential	24 Gy/3 fractions, 50 Gy/4 fractions, 45 Gy/15 fractions	pembrolizumab	19.20 months	41.7

SBRT, stereotactic body radiation therapy; GM-CSF, granulocyte-macrophage colony-stimulating factor; NR, not reached.

**Table 2.** Ongoing clinical trials evaluating abscopal responses

Trial	Tumor type	Systemic therapy	RT	Phase	Sequence of systemic therapy

NCT01976585	low-grade lymphoma	intratumoral Flt3L and poly-ICLC	low dose	1/2	–
NCT02768558	lung cancer	sequential nivolumab, concurrent cisplatin + etoposide	60 Gy	3	concurrent followed by sequential
NCT03192059	cervical and uterine cancer	pembrolizumab	8 Gy/3 fractions	2	concurrent
NCT02992912	multiple	atezolizumab	45 Gy/3 fractions	2	concurrent
NCT04245514	stage III lung cancer	neoadjuvant and adjuvant durvalumab, neoadjuvant cisplatin + docetaxel	20 × 2 Gy every day for 4 weeks, 5 × 5 Gy every day for 1 week, or 3 × 8 Gy every other day for 1 week	2	neoadjuvant followed by adjuvant
NCT03721341	multiple (4–10 lesions)	palliative RT, chemotherapy, immunotherapy, hormone therapy, or observation	SABR (20 Gy/1 fraction, 30 Gy/3 fractions, or 35 Gy/5 fractions) along with investigator-chosen therapy, including chemotherapy, immunotherapy, hormone therapy, and active surveillance	3	concurrent
NCT03137771	non-small cell lung cancer	docetaxel, erlotinib, gemcitabine, pemetrexed ± pembrolizumab	SBRT	N/A	sequential
NCT04785287	multiple	BMS-986218 ± nivolumab	SBRT induced RadScopal effect	1/2	concurrent
NCT05039632	multiple	intratumoral NBTXR3 + ipilimumab + nivolumab	abscopal versus RadScopal effect	1/2	sequential
NCT03480334	relapsed Hodgkin’s lymphoma	nivolumab	20 Gy	2	concurrent
NCT04873440	multiple	inhaled manganese	conventional RT vs. SBRT	1/2	sequential
NCT04238169	advanced NSCLC	toripalimab vs. bevacizumab + toripalimab	SBRT 30–50 Gy/5 fractions (2–4 lesions)	2	concurrent
NCT03449238	breast cancer with brain metastases	pembrolizumab	SRS	1/2	concurrent
NCT03457948	NET and metastatic liver lesions	pembrolizumab	PRRT using 177Lu-DOTA0-Tyr3-octreotate vs. transarterial embolization vs. yttrium-90 microsphere radioembolization	2	concurrent

Poly-ICLC, polyinosinic-polycytidylic acid-poly-*l*-lysine carboxymethylcellulose; Flt3L, FMS-like tyrosine kinase 3 ligand; SABR, stereotactic ablative radiotherapy; NET, neuroendocrine tumor; SBRT, stereotactic body radiation therapy; PRRT, peptide receptor radionuclide therapy; NSCLC, non-small cell lung cancer; SRS, stereotactic radiosurgery; RT, radiation therapy.

**Table 3.** Outstanding questions on abscopal phenomenon

• Can we prospectively predict and identify trends in the abscopal effect using biomarkers?
• The translational validity of results of preclinical studies of abscopal phenomenon is still underwhelming, so will we have clinical corroboration in prospective studies in the future?
• When and how can we reproduce abscopal effects clinically?
• How can abscopal responses significantly affect patient outcomes?
• Can ARs cross the blood-brain barrier?
• What is the window of opportunity for optimal synthetic lethality of RT and immune agents in eliciting the abscopal effect?
